# Tumor expression of dopamine receptor and prolactin is associated with acute dopamine agonist response in acromegaly

**DOI:** 10.3389/fendo.2026.1930467

**Published:** 2026-10-19

**Authors:** Shahriar Atai, Merisa Abusdal, Maren Ileby Wessel, Olivera Casar-Borota, Kristin Astrid Øystese, Jacob Winther, Jens Bollerslev, Ansgar Heck

**Affiliations:** 1 Section of Specialized Endocrinology, Oslo University Hospital, Oslo, Norway; 2 University of Oslo, Faculty of Medicine, Oslo, Norway; 3 Research Institute of Internal Medicine, Oslo University Hospital, Oslo, Norway; 4 Department of Immunology, Genetics and Pathology, Uppsala University, Uppsala, Sweden; 5 Department of Clinical Pathology, Uppsala University Hospital, Uppsala, Sweden; 6 Department of Medicine, Haukeland University Hospital, Bergen, Norway; 7 Department of Endocrinology, Akershus University Hospital, Lørenskog, Norway

**Keywords:** cabergoline, D2R, dopamine 2 receptor, PitNET, pituitary adenoma, tumour, pituitary neuroendrocrine tumor

## Abstract

**Objectives:**

In acromegaly, primary treatment is usually pituitary surgery. Remission rates are approximately 50% with pituitary tumor size and invasiveness playing important roles. Medical therapy is the secondary treatment for many patients. Yet, dopamine agonist responsiveness is variable, and reliable prognostic markers are lacking. We aimed to identify markers of dopamine agonist responsiveness. We hypothesized that (i) dopamine agonist response correlates with high dopamine receptor D2 expression and prolactin expression and secretion and (ii) estrogen receptor α expression is associated with tumor properties.

**Design:**

Single-center retrospective cohort analysis with treatment-naive patients who had undergone a bromocriptine test as a marker for dopamine agonist response.

**Methods:**

Sixty-nine patients with newly diagnosed acromegaly between 2008 and 2017 were included. During this period, the bromocriptine test was performed routinely at our center. Baseline hormone concentrations and MR images were evaluated retrospectively. Immunohistochemical staining of hormones and receptors and gene expression of dopamine receptor D2 in tumor tissue were performed and assessed.

**Results:**

Dopamine agonist test response and high dopamine receptor D2 gene expression were correlated (*r* = 0.53; *p* ≤ 0.001), and both were associated with higher serum prolactin concentration and tumor prolactin expression, assessed by immunohistochemistry. Prolactin and estrogen receptor α expression were correlated, and strong expression was associated with smaller and less invasive tumors.

**Conclusions:**

Dopamine receptor D2 and prolactin expression are markers for dopamine agonist test response, providing valuable insights for personalized management and optimization of medical therapy in patients with acromegaly. Although prolactin and estrogen receptor α expression may be associated with tumor growth, the clinical applicability remains unclear.

## Introduction

According to the 2022 World Health Organization (WHO) classification, several subtypes of pituitary somatotroph tumors can cause acromegaly, each with distinct characteristics regarding tumor behavior, hormone secretion, and histopathological attributes (1, 2). The granulation pattern defined by cytokeratin (CK) expression is emphasized. Densely granulated (DG) tumors are usually smaller and exhibit more hormone secretion compared with sparsely granulated (SG) tumors (3, 4).

Dopamine agonists (DAs) such as cabergoline and bromocriptine (BC) are effective for the treatment of prolactinomas and may reduce growth hormone (GH) secretion and tumor size in somatotroph tumors, though less frequently than for prolactinomas (5–7). Treatment recommendations suggest using DAs in cases with mildly elevated insulin-like growth factor 1 (IGF1) levels without further guidance (8, 9). Recent studies indicate that GH and prolactin co-secretion is common in acromegaly with cabergoline being more effective for this subgroup (5, 10). The expression of dopamine receptor D2 (DRD2) may correlate with the effect of DAs (6, 11). Activation of DRD2 reduces hormone release and may induce apoptosis (7). DRD2 is described to have two isoforms where activation of the short isoform seems to have a stronger effect (12, 13). However, immunohistochemistry (IHC) of DRD2 is not performed in clinical routine, and the *in vivo* relation between DRD2 expression and treatment response remains unclear. Reliable markers for DA responsiveness are lacking, and there is a need to further individualize treatment of patients with acromegaly (5, 14, 15).

A standard low-dose [2.5 mg] BC test has historically been part of the baseline evaluations of patients with acromegaly. The test may predict long-term DA effectiveness by lowering GH levels (16–19) and may be correlated with baseline prolactin levels (20). The association between the BC test and DRD2 expression is unexplored, raising questions about its usefulness in individualizing DA treatment.

Estrogen receptor alpha (ERα) is expressed in the pituitary gland (21) including in somatotroph tumors with lactotroph differentiation (1, 2). Low ERα expression is associated with increased aggressiveness and tumor growth in non-functioning pituitary tumors and prolactinomas (22, 23). Estrogen has been used to treat acromegaly by lowering IGF1 (24). Furthermore, the DA effect on the DRD2 may be estrogen-dependent (13). However, the association between ERα and DA responsiveness as well as somatotroph tumor growth remains unclear.

Four somatostatin receptor (SSTR) subtypes are expressed in the pituitary gland and in pituitary tumors (25). Though interactions between SSTRs and DRD2 have been suggested, they remain unproven in somatotroph tumors (6). Recent studies indicate that prolactin and GH co-secreting tumors express more DRD2 and SSTR1 (11), and low SSTR1 expression is associated with increased GH secretion per tumor volume (26), suggesting an association between SSTR1, DRD2, and prolactin levels.

The aim of this study was to identify markers for DA responsiveness in patients with newly diagnosed acromegaly. The primary outcome was GH decline during the BC test as a surrogate marker for DA response. Furthermore, we aimed to explore how histological tumor characteristics serve as markers for DA response. We hypothesized that:

- DA responsiveness correlates with DRD2 expression (primary hypothesis).
- DA responsiveness correlates with prolactin expression and secretion.
- There is an association between SSTR1 and DRD2 to DA responsiveness.
- ERα correlates with DA responsiveness and tumor properties.

## Materials and methods

### Patients

Between 2008 and 2017, the BC test was performed as part of the routine clinical workup in patients with newly diagnosed acromegaly. These consecutive patients were identified through our pituitary registry and included in the cohort.

### Baseline serum hormones and tests

At baseline, serum hormone samples, including GH, prolactin, and IGF1, were drawn in the morning after overnight fasting. GH and IGF1 were measured by immunoassays (Siemens IMMULITE, 2000). Two standard calibrators for GH, IS 80/505 (2.4 mU/L = 1 µg/L) and IS 98/574 (3 mU/L = 1 µg/L) were used before and after August 2008, respectively. The standard calibrator for IGF1 was IS 87/518. IGF1 was divided by the age-adjusted upper limit of normal (IGF1/ULN). Prolactin was measured by immunoassay (DELFIA kit, Turku, Finland), standard calibrator IS 84/500.

The BC test was performed as previously described (18, 27). After an overnight fast, three baseline GH samples were drawn 30 min apart and the mean value was calculated. After an oral 2.5 mg BC dose, GH was drawn at 2, 4, and 6 h. As in previous studies, a positive response was defined as >50% reduction in nadir GH concentrations compared with the baseline mean value (27, 28).

### MRI

Assessment of MR images at baseline included tumor size and volume (ellipsoid formula), invasiveness based on the Knosp criteria (29), and T1- and T2-weighted signal intensity.

### Immunohistochemistry

GH, prolactin, thyroid-stimulating hormone (TSH), CK8, ERα, SSTR1, SSTR2, SSTR5, and DRD2 staining was performed at the Department of Pathology, Oslo University Hospital and Uppsala University Hospital, using formalin-fixed paraffin-embedded tissue.

The following antibodies were used at Oslo University Hospital: GH (Dako, Glostrup, Denmark, A0570), prolactin (Dako, Glostrup, Denmark, A0569), TSH (Dako, Glostrup, Denmark, M3503), and CK8 (Zymed, California, USA, 18-0185Z). Since August 2017, CK8 was stained using Epitomics/Abcam, California, USA, AC-0007, clone EP17. If hormone and CK staining were unavailable, the same antibodies were used at Uppsala University Hospital except for prolactin (Cell Marque, California, USA, 210A-14), TSH (Novocastra, Newcastle, UK, NCL-TSH), and CAM 5.2 (CK7/CK8, BD Transduction, California, USA, 345779).

At Uppsala University Hospital, receptors were stained using the following antibodies: DRD2 (polyclonal, Merck Millipore, California, USA AB5084P; monoclonal, Bio-Techne, Minneapolis, USA MAB9266), ERα (Ventana-Roche, Tuscon, USA 790-4324), and SSTRs (SSTR1 ab137083, SSTR2 ab134152, SSTR5 ab109495 from Abcam).

SSTR and DRD2 staining were conducted using Autostainer Link 48; GH, prolactin, TSH, and CAM 5.2 with DAKO Omnis; and ERα with Ventana Roche. Detection was performed with the Envision FLEX kit as per the manufacturer’s instructions. Pancreas tissue served as positive control for SSTR antibodies; prostate/salivary gland for CAM 5.2; pituitary gland tissue for prolactin, TSH, and GH; and Erα-positive breast cancer tissue for the ERα antibody. All control tissues were based on surgical samples.

Digital images of the histological slides were captured at Uppsala University Hospital using a Hamamatsu NanoZoomer whole-slide scanner and assessed using NDP.view2.

### Immunohistochemistry assessment

For the assessment of GH, prolactin, TSH, ERα, and SSTR, immunoreactivity score (IRS) was applied as previously described (26, 30). Prolactin staining was categorized as negative (IRS 0–1) and positive (IRS 2–12) in some of the analyses reflecting clinical pathology routine. CK granulation pattern was categorized as (1) DG, >70% tumor cells with perinuclear staining; (2) SG, >70% tumor cells with fibrous bodies; (3) intermediate-type (<70% cells with perinuclear staining and occasional fibrous bodies); and (4) negative cases with no CK staining (1, 4). IHC slides were assessed by M.A. in collaboration with a senior pathologist (O.C.-B). Both were blinded to clinical data.

### *DRD2* gene expression analysis

Tumor tissue samples were collected and snap-frozen during surgery and stored at −80°C, except for one sample that was embedded in optimal cutting temperature medium. Tumor tissue was homogenized in TRIzol reagent (Life Technologies, Life Technologies, 15596018) using a rotor–stator homogenizer. RNA was extracted using the miRNeasy Mini Kit (QIAGEN, Hilden, Germany, 217004) with DNase treatment (QIAGEN, Hilden, Germany, 79256). RNA quality and concentration were assessed by a NanoDrop ND-1000 Spectrophotometer (NanoDrop Technologies, Wilmington, USA,), resulting in the exclusion of three samples due to low yield. cDNA was synthesized in a Mastercycler X50s (Eppendorf Mastercycler X50, 6311000045) using high-capacity cDNA reverse transcription kit (Applied Biosystems, Waltham, USA, 4368814), with 1,000 µg of RNA. Diluted cDNA (1:10) was loaded with an automated pipetting system (Eppendorf epMotion 5070 CB [RRID: SCR 025064]) for real-time quantitative PCR (RT-qPCR) on the ABI7900 (Applied Biosystems) using PerfeCTa™ SYBR^®^ Green SuperMix (Quantabio, Beverly, USA, 733-1251) with two technical replicates. *DRD2* primer forward and reverse sequences were CAACGGGTCAGACGGGAG and GAATTTCCACTCACCTACCACC. Primers were designed to span multiple exon junctions to avoid gDNA contamination. Threshold cycle (Ct) values were normalized to the geometric mean of the selected reference genes GAPDH and PSMC4 (31). Relative expression levels were calculated as fold-change values (2−^ΔΔCt^) to the cohort mean, with Ct values for *DRD2* ranging from 24.17 to 30.48. Low-quality data were excluded from analyses.

### Statistics

Most variables were not normally distributed. Non-parametric tests were used when at least one variable was not normally distributed. Depending on whether the BC test was defined as categorical (positive or negative) or continuous (0%–100%), different statistical tests were applied. For the prior, Pearson chi-square, Fisher’s exact test, and Wilcoxon rank-sum tests were used. For the latter, Wilcoxon rank sum, Spearman’s rho, and Kendall’s tau test for correlation were used. Gender differences were assessed using the Wilcoxon rank-sum, Pearson chi-square, and Fisher’s exact tests, and partial correlation analyses were performed using Kendall’s tau, with analyses adjusted for gender.

For IHC IRS, Wilcoxon rank sum and Kendall’s tau were performed. For prolactin staining defined as positive or negative, the Wilcoxon rank-sum and Fisher’s exact tests were performed. For granulation pattern, Fisher–Freeman–Halton and Kruskal–Wallis tests were used, the latter supplemented with Dunn’s test to explore the correlation between categories. For gender differences, the Wilcoxon rank-sum, Fisher–Freeman–Halton, and partial correlation analyses with Kendall’s tau were used, with the latter adjusted for gender.

For *DRD2* gene expression, the Wilcoxon rank-sum, Spearman’s rho, and Kendall’s tau tests were performed. Partial correlation analyses were performed to adjust the correlations between the BC test and *DRD2* for age, gender, tumor volume, prolactin, and GH concentration and expression (Spearman and Kendall). Similar partial correlation analyses were performed for the BC test and prolactin concentrations and prolactin IHC expression adjusted for *DRD2.*

Dichotomization of the GH reduction after the BC test was chosen as a secondary statistical outcome to give a clinically applicable overview of the cohort and the differences between responders and non-responders.

RStudio version 4.3.0 was used for statistical analyses and graph design. A *p*-value <0.05 was considered significant.

### Ethics

The study was approved by the Regional Ethical Committee of the South-East health region of Norway (REK, number 15240), and all patients gave written informed consent.

## Results

### Baseline characteristics

Of 141 consecutive patients diagnosed with acromegaly between 2008 and 2017, 71 underwent a BC test. In 70, the BC test was not performed or completed due to logistical reasons or contraindications to DA (*n* = 56), acute surgery due to cranial nerve affection (*n* = 5), hypotension or syncope during test (*n* = 5), DA treatment before test (*n* = 2), lactose intolerance (*n* = 1), and pituitary apoplexy (*n* = 1). Two patients were excluded: one was lost to follow-up shortly after diagnosis, and the other did not provide consent. Thus, 69 patients with the BC test were included in the retrospective analyses. The baseline characteristics of the included patients are presented in **Table 1**.

**Table 1:** Baseline characteristics.

Median (IQR)	Positive BC test, *n* = 42	Negative BC test, *n* = 27	Total, *n* = 69	*p*-value
Female/male, *n*	16/26	15/12	31/38	0.16
Age, years	45 (31–54)	52 (43–59)	46 (37–56)	0.06
Follow-up time, years	8 (7–10)	8(7–10)	8 (7–10)	
GH [µg/L]	8 (5–19)	9 (5–19)	8 (5–20)	0.99
IGF1/ULN	2.6 (1.8–3.5)	3.4 (2.8–3.8)	3.0 (2.1–3.6)	0.05
Prolactin [µg/L]	20 (13–39)	11 (9–22)	17 (10–33)	0.02
Prolactin > ULN/<ULN, *n*	23/19	6/21	29/40	0.01
BC test % GH reduction	74% (64%–83%)	37% (27%–43%)	59% (42%–78%)	
Macro/microadenoma, *n*	32/10	22/5	54/15	0.6
Tumor volume, mL	0.8 (0.4–2.3)	1.1 (0.6–2.7)	1.0 (0.4–2.4)	0.54
Knosp 0–1/2–4, *n*	31/11	13/13	44/24	0.05
T2 hypo/other	25/17	16/10	41/27	0.86

The baseline characteristics stratified into positive or negative bromocriptine (BC) test with *p* describing their statistical differences. In one patient with a negative BC test, tumor volume, invasiveness, and intensity were not possible to assess. *p* < 0.05 was considered significant.

### Determinants of the BC test response

There were no significant sex differences in baseline characteristics between patients with positive versus negative BC tests. Patients with a positive test had a lower IGF1/ULN and invasiveness defined as tumor Knosp grade and higher prolactin concentrations (**Table 1**). GH reduction after the BC test was more pronounced in lower age (*r* = −0.27, *p* = 0.03) and higher prolactin concentrations (*r* = 0.24, *p* = 0.05).

#### *DRD2* expression

DRD2 IHC analysis was unsuccessful. Both antibodies tested on postmortem central nervous system (CNS) tissue showed distinct intracellular granular labeling; however, background staining was high and could not be reduced. Tests on surgical specimens from CNS biopsies and pituitary glands yielded only diffuse staining without specificity.

Gene expression analyses were available in 54 patients. Higher *DRD2* expression was seen in better DA response in the BC test (*r* = 0.53, *p* < 0.001; **Figure 1A**) and a positive BC test (*p* = 0.001). The association between the BC test and *DRD2* remained significant when adjusted for gender, age, tumor volume, GH and prolactin concentrations, and staining (*p* ≤ 0.002 after adjustments). Higher *DRD2* expression was seen in patients with higher prolactin concentrations (*r* = 0.41, *p* = 0.002), prolactin IHC expression (*r* = 0.38, *p* < 0.001, and *p* = 0.002 for prolactin expression analyzed as binary variable; **Figure 1B**), and lower IGF1/ULN (*r* = −0.27, *p* = 0.05). The correlation between the BC test and prolactin concentrations and prolactin IHC expression did not remain statistically significant when adjusted for *DRD2* (*r* = 0.05, *p* = 0.72 and *r* = 0.14, *p* = 0.14).

**Figure 1:**
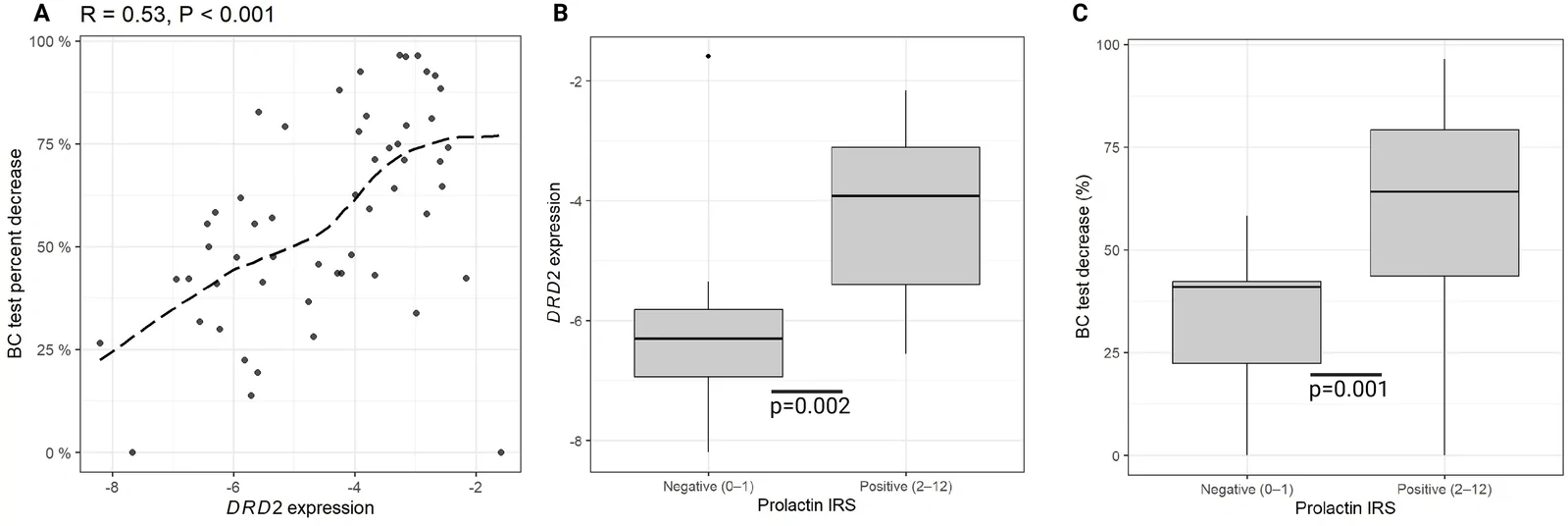
Correlations between DRD2 gene expression, the BC test, and prolactin expression. **(A)** Scatter plot with LOESS curve for data visualization. Spearman correlation between DRD2 expression and the BC test: r = 0.53; *p* < 0.001; n = 54. **(B)** Boxplot showing DRD2 expression when prolactin staining was categorized as positive or negative. **(C)** Boxplot showing percentual decrease in the BC test when prolactin staining was categorized as positive or negative. **(B, C)** n = 57 for positive and n = 9 for negative prolactin expression.

#### IHC hormone and receptor staining

Sixty-six samples were available for hormone and 56 for receptor analyses, with the results of the assessments summarized in **Table 2**. All somatotroph tumors expressed GH except for one, and 57 of 66 expressed prolactin. There were no significant sex differences in the expression of hormones and receptors, except for a trend towards higher ERα expression in men (*p* = 0.06).

**Table 2:** IHC tumor expression of hormones based on IRS and differences between BC test positive and negative patients.

IRS median (IQR)	Total	BC test positive	BC test negative	*p*-value
GH	11 (6–12)	9 (5–12)	12 (6–12)	0.73
Prolactin	3 (2–6)	4 (3–6)	3 (1–6)	0.04
TSH	0 (0–0)	0 (0–0)	0 (0–0)	0.53
ER	2 (1–4)	2 (2–4)	2 (1–3)	0.21
SSTR1	2 (1–2)	2 (1–2)	2 (1–2)	0.45
SSTR2	12 (8–12)	12 (9–12)	12 (8–12)	0.92
SSTR5	9 (6–12)	9 (6–12)	12 (8–12)	0.06

*n* = 66 for hormones (positive BC test *n* = 39, negative BC test (*n* = 27) and *n* = 56 for receptors (positive BC test *n* = 31, negative BC test *n* = 25). DRD2 staining was not successful. *p* < 0.05 was considered significant.

Prolactin expression was correlated with better DA response in the BC test (*r* = 0.21, *p* = 0.02), in addition to the binary BC test outcome (*p* = 0.04) with no sex differences. We found no correlation between the BC test and the expression of GH, TSH, ERα, SSTR1, SSTR2, or SSTR5 (**Table 2**).

Higher prolactin expression by IHC was found in patients with higher serum prolactin concentrations (*r* = 0.40, *p* < 0.001), higher *DRD2* gene expression (*r* = 0.38, *p* < 0.001), higher ERα expression (*r* = 0.44, *p* < 0.001), lower tumor volume (*r* = −0.25, *p* = 0.01), and lower Knosp grade (*p* = 0.04). These correlations remained significant when dichotomizing prolactin IRS into negative (*n* = 9) and positive (*n* = 57) staining. **Figure 1C** describes positive and negative prolactin staining and the BC test outcome. Patients with negative prolactin staining tumors were older (*p* = 0.002) and had higher IGF1/ULN (*p* = 0.04). When both prolactin staining and the BC test were defined as binary variables, they were associated (phi = 0.39, *p* = 0.002) (**Table 3**). When we defined the test response as outcome and prolactin staining as the marker as in **Table 3**, we found a sensitivity of 97% (95% CI 87%–100%), specificity of 30% (95% CI 14%–50%), positive predictive value (PPV) of 67% (95% CI 61%–72%), and negative predictive value (NPV) of 89% (95% CI 51%–98%). **Figure 2** demonstrates prolactin staining in four patients.

**Table 3:** Correlation between prolactin staining and the BC test.

IHC staining	Positive BC test	Negative BC test	Total *n*
Prolactin +	38	19	57
Prolactin −	1	8	9
Total n	39	27	66

Phi = 0.39; *p* = 0.002.

**Figure 2:**
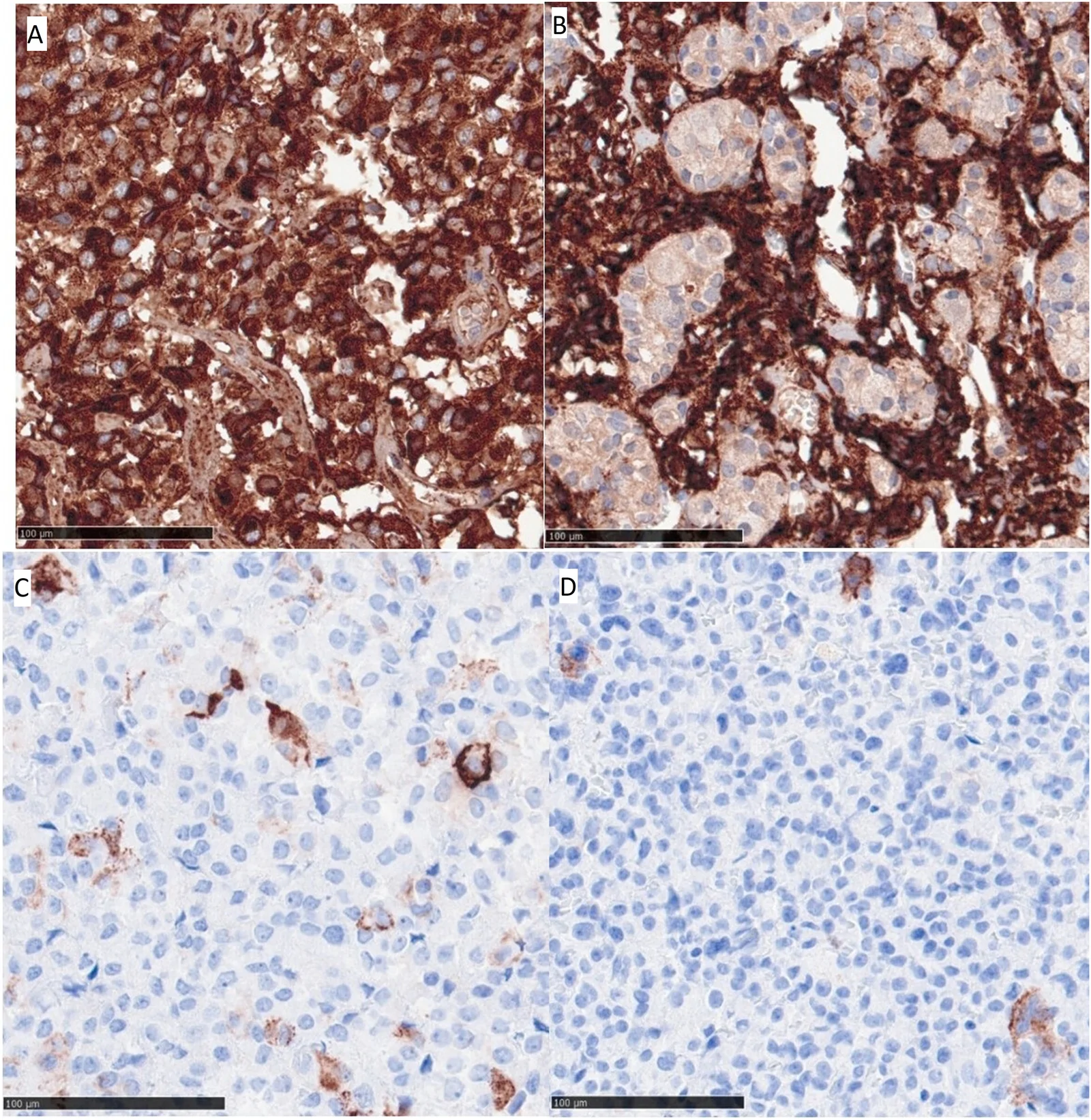
Prolactin staining in four selected patients. IRS of 12 **(A)**, 8 **(B)**, 3 **(C)**, and 1 **(D)**.

Higher ERα expression was associated with higher prolactin expression (*r* = 0.44, *p* < 0.001), prolactin concentrations (*r* = 0.18, *p* = 0.07), lower tumor volume (*r* = −0.2, *p* = 0.04), and lower Knosp grade (*p* = 0.02). There was no difference in ERα expression in women below or above 50 years of age.

#### Granulation pattern

The proportion of subgroups in the different granulation patterns was consistent with other studies (3, 4, 32, 33). Our data on granulation patterns are presented in the **Supplementary File**.

## Discussion

In patients with newly diagnosed acromegaly, a better DA response to the standard BC test was found in patients with high tumor *DRD2* gene expression. Both DA test response and DRD2 gene expression correlated with stronger prolactin IHC expression and elevated baseline serum prolactin concentrations. DA-responsive patients had less invasive tumors. Prolactin and ERα expression were associated, and strong expression was associated with smaller and less invasive tumors. We did not find an association between SSTR1 staining and *DRD2* gene expression or DA test response.

Our study demonstrated that DA responsiveness assessed by a low-dose BC test correlates with high *DRD2* gene expression. The acute BC test was mainly reported in the 1970s and 1980s for prediction of long-term effects of DA in patients with acromegaly (16–20, 27, 28). Previous studies on the *in vivo* expression of DRD2 correlated with DA effects are limited. *In vitro* studies in somatotroph cells have demonstrated that *DRD2* expression correlated to the response to cabergoline (trend) (11) and to quinagolide (34). A recent clinical study found that low *DRD2* gene expression had a high negative predictive value on the effects of cabergoline (35). However, *DRD2* gene expression is not available as clinical routine assessment. As in our study, the lack of reliable antibodies against DRD2 for clinical-pathologic IHC remains a challenge. The poor antibody reliability and specificity may have several reasons, including different receptor isoforms (6, 13). Detection of protein expression by IHC could improve the understanding of DRD2 function, isoforms, intracellular mechanisms of action, and the effects of DAs.

We found that DA test response was associated with stronger tumor prolactin expression and higher serum prolactin concentrations. A recent study suggested cabergoline as part of the first-line medical treatment in patients with prolactin co-secretion (10). However, the risk of confounding was high due to the retrospective study design. Evidence from well-powered prospective controlled studies is lacking (36). A large controlled trial of DA treatment was published almost 30 years ago (37) and in more recent studies (5, 10); however, somatotroph tumors were categorized mainly by hyperprolactinemia and not histologically.

In our cohort, most somatotroph tumors (86%) had tumor prolactin expression. This proportion is considerably higher than that reported in previous studies showing hyperprolactinemia in 20%–30% of patients with acromegaly (5, 10, 37). The discrepancy may be explained by recent findings showing that 41% of acromegaly patients without hyperprolactinemia had positive prolactin staining by IHC (38). Notably, no consensus exists on the definition of prolactin co-secreting somatotroph tumors, and the WHO classification emphasizes a histological classification (1). It is not clear how prolactin expressing somatotroph tumors responds to DAs. A recent *in vitro* study showed that octreotide and cabergoline had similar effects in reducing GH in GH- and prolactin-co-secreting cell cultures where most samples expressed prolactin (11). In line with our results, a prospective study from 1997 evaluated the effects of different DAs in acromegaly and concluded that there was a strong correlation between prolactin staining and normalization of GH excess (39). A small prospective study found normalization of IGF1 in patients who received add-on therapy with cabergoline with a positive prolactin staining, with fewer than half having hyperprolactinemia (40). A prospective study exploring the role of cabergoline as an addition to somatostatin receptor analogues concluded that the additive effect was regardless of prolactin expression. However, IHC was only available in 15 of 52 patients, and assessment methods were not described (41). Other prospective studies found no correlation between the prolactin-expressing tumors and the effects of cabergoline, neither as mono- nor add-on therapy (42–45). In conclusion, previous studies of histological prolactin expression have yielded conflicting results, with a small number of IHC samples and unspecified assessment methodology. Moreover, a meta-analysis published more than a decade ago concluded that DAs may have beneficial effects in some patients with acromegaly and normal prolactin levels (46).

Our finding of a correlation between *DRD2* gene expression and DA test response was independent of GH and serum prolactin concentration and tumor expression. When adjusted for *DRD2*, the correlation between prolactin and the BC test was no longer present. This implies that the DA effect is mediated through the DRD2, as expected, and that prolactin expression may serve as an indirect marker for DRD2 expression and DA responsiveness.

The WHO classification discriminates pure somatotroph tumors (GH positive) from tumors with both somatotroph and lactotroph differentiation (GH, prolactin, and ERα positive) (1, 2). We found a correlation between ERα and prolactin expression, and both were seen in small tumors with low invasiveness. Multiple studies have assessed the associations between ERα expression and somatotroph tumor characteristics, but the results are conflicting (10, 38, 47, 48). A recent study in patients with acromegaly did not find an association between ERα expression and tumor size (47). One study divided somatotroph tumors into GH or GH + prolactin secreting and did not find a significant difference in tumor volume (10). Another large multicenter study defined patients into the same groups and did not find a difference in invasiveness, and tumor volume was not assessed (38). A similar study did not find a significant difference in tumor volume in acromegaly patients with or without hyperprolactinemia (48).

The strengths of our study are the large and well-characterized cohort with standardized dynamic *in vivo* test supported by *DRD2* gene expression and complementary IHC analyses. Routine baseline testing as described in our study is less prone to confounding than registry data of clinical DA treatment in previous clinical studies.

The lack of reliable antibodies for IHC DRD2 detection is a limitation that we sought to solve by gene expression analysis. We did not distinguish between the long and short isoforms of *DRD2*, which may have different mechanisms of action and effects (12, 13, 49–51). Gene expression does not necessarily reflect protein expression, function, or cellular localization. We used the BC test as a surrogate measure for DA response. Although it cannot replace studies of the long-term effect of DAs, the BC test is an easily accessible tool to study potential markers for DA response. The 70 patients who did not perform a BC test between 2008 and 2017 had lower prolactin concentrations and GH excess, which may have introduced bias, although other differences were not found including age, sex, and tumor size. Remission after surgery in acromegaly is approximately 50% with tumor size and invasiveness being the most contributing factors (52–54). Although not clinically applicable, our findings of a correlation between high tumor ERα and prolactin expression and low tumor volume and invasiveness may be of interest and needs to be further elucidated in future studies.

In conclusion, we demonstrated that easily available histological analyses with prolactin staining may add valuable information for the individualized pharmaceutical management of patients with acromegaly. *DRD2* gene expression analyses support our results. We found a correlation between ERα and prolactin tumor expression and low tumor volume and invasiveness; however, the clinical applicability of these findings remains unclear.

## Data Availability

The datasets generated and analyzed for the current study are not publicly available because of privacy and regulatory constraints. However, data may be made available from the corresponding author upon reasonable request, subject to applicable laws, regulations, and ethical approvals.
