# Supplementary material for: Tumor expression of dopamine receptor and prolactin is associated with acute dopamine agonist response in acromegaly
Source: Front Endocrinol (Lausanne). 2026 Oct 19;17:1930467. doi: 10.3389/fendo.2026.1930467 (PMC13724516; doi:10.3389/fendo.2026.1930467)
Supplement: Supplementary file 1 [file Supplementaryfile1.docx]

# Supplementary material

## Granulation pattern

We found 37 densely granulated (DG) (58%), 12 sparsely granulated (SG) (18.5%), ten intermediate (15.5%) and five (8%) who did not stain for cytokeratin (CK) in 64 patients where CK staining was available. Most patients with DG and intermediate tumors had a positive BC test compared to SG tumors who more frequently had a negative test (p=0.003). The intermediate group expressed more prolactin than DG and SG, and no difference was found between DG and SG. There were no differences in the dopamine receptor D2 gene expression or ERα staining between the categories.

Our study showed that intermediate granulation pattern had the strongest prolactin expression and favorable response to dopamine agonist. Studies have shown similar characteristics in DG and intermediate tumors (1, 2), as our study partially did, with WHO describing them as indistinguishable (3, 4). The proportions of subgroups were consistent with previous studies (1, 5). The five cases who did not stain for CK had no specific characteristics, as in other studies (5, 6).

## References

1.	Obari A, Sano T, Ohyama K, Kudo E, Qian ZR, Yoneda A, et al. Clinicopathological features of growth hormone-producing pituitary adenomas: difference among various types defined by cytokeratin distribution pattern including a transitional form. Endocr Pathol. 2008;19(2):82–91. doi: 10.1007/s12022-008-9029-z. **Reference 4 in the article**

2.	Fougner SL, Casar-Borota O, Heck A, Berg JP, Bollerslev J. Adenoma granulation pattern correlates with clinical variables and effect of somatostatin analogue treatment in a large series of patients with acromegaly. Clin Endocrinol (Oxf). 2012;76(1):96–102. doi: 10.1111/j.1365-2265.2011.04163.x. **Reference 33 in the article**

3.	Lopes MB, Asa SL, Kleinschmidt-DeMasters BK, Mete O, Osamura RY, C. V. Central Nervous System Tumours. 5 ed: WHO; 2021. 9 p. **Reference 1 in the article**

4.	Asa SL, Mete O, Perry A, Osamura RY. Overview of the 2022 WHO Classification of Pituitary Tumors. Endocr Pathol. 2022;33(1):6–26. doi: 10.1007/s12022-022-09703-7. **Reference 2 in the article**

5.	Swanson AA, Erickson D, Donegan DM, Jenkins SM, Van Gompel JJ, Atkinson JLD, et al. Clinical, biological, radiological, and pathological comparison of sparsely and densely granulated somatotroph adenomas: a single center experience from a cohort of 131 patients with acromegaly. Pituitary. 2021;24(2):192–206. doi: 10.1007/s11102-020-01096-2. **Reference 32 in the article**

6.	Heck A, Emblem KE, Casar-Borota O, Bollerslev J, Ringstad G. Quantitative analyses of T2-weighted MRI as a potential marker for response to somatostatin analogs in newly diagnosed acromegaly. Endocrine. 2016;52(2):333–43. doi: 10.1007/s12020-015-0766-8. **Reference 3 in the article**
